# Notes and News

**Published:** 1901-08-10

**Authors:** 


### Notes and News.

The National Association of Teachers of the Deaf held their biennial conference at Oxford last week. The conference passed a resolution in favour of the extension of Government aid to deaf and blind children in Ireland.

The late Mr. Samuel Dobbs, of West Hampstead and Princes Street, has bequeathed £3,150 to the North London Hospital for Consumption, and the residue of his estate, valued at upwards of £16,000, to the Westminster Hospital.

A new wing has been added to the North Ormesby Cottage Hospital, and named the Swan Memorial Wing, in memory of the late Captain Swan, who was treasurer of the institution. The cost of the new wing amounted to about £5,500, and it will be utilised for administrative purposes.

The crusade against spitting is being taken up in Germany. Prevention is better than cure, but it does not seem to have occurred to the hygienic advocates that the chief people to enlist are those who have the charge of the up-bringing of young people. Spitting is mostly a matter of habit, acquired especially in the case of boys by emulating the manly habits of their elders.

The first Egyptian Congress of Medicine is to be held in December, 1902, under the patronage of the Khedive. The work of the Congress will be principally devoted to affections peculiar to Egypt, and a preliminary programme has already been arranged on which a good number of European names appear, and the range of discussion is very comprehensive. The Secretary-General is Dr. Voronoff, who writes from Cairo that he will be glad of the co-operation of European medical men.

A conversazione was held last week at the new Dental Hospital of London in Leicester Square. The guests were received by members of the medical staff, and the prizes were distributed to the students by Mr. T. Arnold Rogers, the senior consulting surgeon. The Dean, in his speech, said that the new hospital largely owed its existence to the energy of Mr. Morton Smale. The Dean and Mr. Lloyd Williams paid a visit to the United States to study the latest developments in dental teaching before the new premises were completed.

The people of Cambridge have decided to endeavour to erect a new wing at the Addenbrooke's Hospital as a local memorial to Queen Victoria. The sum required is £5,000.

The new Workhouse Infirmary at Guildford has been opened by Mr. J. Deverell, the Chairman of the Board of Guardians. There is accommodation for 60 patients. The architect is Mr. Percy Adams, and the total cost has been £10,450.

The present report of health from Belfast shows an unfortunate return of typhoid fever. In spite of the attention drawn during the last and recent epidemic to the insanitary condition of many parts of the city the defects are still of a glaring nature.

A question having been asked in Parliament as to whether a Royal Commission will inquire into the question of the transmission of bovine tuberculosis to the human species, Mr. Long has replied that the matter is under consideration.

An extraordinary complaint has been made by the employees at the New Jersey State Hospital, that they and the patients are practically fed on tinned meat and vegetables. Nothing could be more easily refuted than such a charge, which should be met by the authorities in a very definite manner.

The annual meeting of the British Dental Association was held in the examination hall of the Royal College of Physicians and Surgeons, on the Victoria Embankment, from the 2nd to the 6th inst., and the first general meeting of the International Dental Federation was held on Wednesday, August 7th, at Cambridge.

New York during the present summer has been visited with an unusual amount of both smallpox and pneumonia, diseases which show themselves there as a rule during the winter months. Mosquitos have been more than usually numerous during the same season, so that with the great heat which has prevailed, New York has fared very badly.

The Society of Apothecaries has successfully sued a chemist at Carmarthen who had practised as an apothecary without a certificate. The chemist was in the habit of prescribing for customers to his shop, and had more than once been warned by the coroner at various inquests previous to the prosecution. The poor are always willing to receive medical advice from chemists, and their readiness to follow treatment so prescribed is a danger from which it is necessary to protect them as far as possible.

At the meeting of the British Medical Association, Dr. Patrick Manson received the Stewart prize, which is awarded biennially for research in the origin and prevention of epidemic disease. The Committee of Selection made the award in the following terms: The Committee recommend that the Stewart prize be awarded to Patrick Manson, C.M.G., M.D., F.R.S., for his researches in the pathology of tropical diseases, especially in regard to the malaria of man and to the life history of the malaria parasite, both in man and the mosquito, and in recognition of the stimulating influence which he has exerted for many years on the study of tropical diseases in the British Empire.

